# Imaging Isomers on a Biological Surface: A Review

**DOI:** 10.5702/massspectrometry.A0078

**Published:** 2019-12-27

**Authors:** Britt S. R. Claes, Emi Takeo, Eiichiro Fukusaki, Shuichi Shimma, Ron M. A. Heeren

**Affiliations:** 1 Maastricht MultiModal Molecular Imaging (M4I) Institute, Division of Imaging Mass Spectrometry (IMS), Maastricht University; 2 Department of Biotechnology, Graduate School of Engineering, Osaka University

**Keywords:** mass spectrometry imaging, isomers, biological surface, derivatization, instrumentation

## Abstract

Mass spectrometry imaging is an imaging technology that allows the localization and identification of molecules on (biological) sample surfaces. Obtaining the localization of a compound in tissue is of great value in biological research. Yet, the identification of compounds remains a challenge. Mass spectrometry alone, even with high-mass resolution, cannot always distinguish between the subtle structural differences of isomeric compounds. This review discusses recent advances in mass spectrometry imaging of lipids, steroid hormones, amino acids and proteins that allow imaging with isomeric resolution. These improvements in detailed identification can give new insights into the local biological activity of isomers.

## INTRODUCTION

Mass spectrometry imaging (MSI) is a label-free analytical technique that allows detection, mapping and identification of many molecules in complex, (biological) sample surfaces in one single experiment.^1,2)^ The possibility to obtain the location of compounds in tissue is of enormous value, as the distribution of compounds is an important piece of information in many areas of biological research.^3)^ With MSI, single mass spectra are recorded at specific *x* and *y* coordinates across the sample. Using dedicated software, images can be generated by selecting peaks of interest in the overall spectrum. Depending on the intensity of these peaks throughout the sample, intensity maps of the selected ions can be visualized.

Tandem mass spectrometry (MS/MS) can support the identification of these compounds. However, when structural isomers have identical fragmentation pathways, then, even with high-mass resolution, mass spectrometry alone cannot distinguish these isomers under conventional ion activation conditions. Nevertheless, resolving these structural isomers is essential for a better understanding of the nature of disease. For example, there is evidence that the dysregulation of pathways involving phospholipase A2 enzymes, which are responsible for the hydrolysis of acyl chains connected to the *sn*-2 position on the glycerol backbone of lipids, are involved in cancer.^4)^ Also for understanding biological processes, it is important to be able to distinguish between these compounds. For instance, testosterone and dehydroepiandrosterone, an intermediate of testosterone, are structural isomers.^5)^ In order to understand the process of spermatogenesis, these isomers require discrimination. Furthermore, chirality is a key characteristic of the function of a compound. Enzymes often display differences in substrate selectivity, and therefore particular enantiomers are utilized in biochemical processes. This is also the case with amino acids, where for example L-amino acids are used as a building block for proteins, but D-amino acids cannot be incorporated.^6)^ Aside from the sequence of the amino acids, also protein identification remains a challenge in MSI due to the various proteoforms and possible post-translational modifications (PTMs).

While these structural differences are subtle, recent developments have made it possible to perform MSI with isomeric resolution for certain molecular classes. This allows us to get new insight into the local biological activity of these isomers. In this review, first, different surface sampling technologies and mass spectrometry (MS) systems within MSI for biological applications will be discussed. Then, the recent advances in isomeric MSI are described with a focus on isomeric imaging of lipids, steroid hormones, amino acids and proteins in tissue sections, as these molecular classes have demonstrated to have relevant diagnostic information but are prone to isomer formation.

## SURFACE SAMPLING AND IONIZATION

MSI can be conducted with various surface sampling technologies based on different ionization methods. The most commonly used are matrix-assisted laser desorption/ionization (MALDI), secondary ion mass spectrometry (SIMS), desorption electrospray ionization (DESI) and liquid microjunction techniques such as liquid extraction surface analysis (LESA) with nano-electrospray ionization (nano-ESI).^7)^

### MALDI

MALDI is one of the main ionization techniques used in MSI. In MALDI, the matrix is of crucial importance.^8)^ The ionization efficiency of different classes of molecules is greatly influenced by the type of matrix used.^9)^ The matrix application on tissues determines the extraction of the surface biomolecules. Subsequently, the solvent from the matrix solution evaporates, causing the crystallization of the matrix mixed with the extracted analyte molecules. When the crystals are irradiated with the pulsed laser light of the MALDI source, the laser energy is absorbed by the matrix molecules and facilitate the explosive desorption and ionization.^1)^ Commercial MALDI MSI instruments allow acquisition rates up to 50 pixels/second and spatial resolutions down to 10 μm,^10)^ while experimental instruments are approaching 147 pixels/seconds,^11)^ or a spatial resolution of 1.4 μm.^12)^

### SIMS

SIMS is one of the oldest desorption/ionization techniques used for MSI, which uses a primary ion beam to ablate material and produce secondary ions.^1)^ The energy of the primary ion beam is higher than the energy by the laser beam during MALDI experiments. Hence, SIMS often yields extensive fragmentation of molecules, limiting the mass range to small molecules. Because of the impact of the primary ions, the kinetic energy causes the release of analyte molecules and ions from the surface.^13)^ Different primary ion beams can be used to change the impact area, known as the damage cross-section, or to decrease fragmentation. The extensive fragmentation has been partly addressed by the development of polyatomic primary beams.^1)^ By the implementation of soft sputtering beams such as (H_2_O)^+^_1000_ or Ar^+^_1500_, thin layers of the samples can be removed, allowing depth profiling and the generation of 3D imaging maps.^14,15)^ Ion beams can be focused with much higher precision than a laser beam, making SIMS a unique tool for high spatial resolution MSI providing a spatial resolution as low as 50 nm with multiplexed ion beam imaging.^16)^

### DESI

DESI is an ambient ionization method that allows direct analysis of samples such as tissue sections, often without any sample preparation.^9,17)^ The soft ionization is based on ESI-like processes.^1)^ By using a charged electrospray solvent mist, molecules are desorbed from the sample surface.^18)^ Similar to ESI, ions are formed by either ion emission or evaporation of neutral solvent molecules.^1)^ To increase the ionization efficiency of the analytes of interest, different solvents can be used in DESI imaging.^9)^ In addition, with DESI it is possible to perform *in situ* derivatization, known as reactive DESI, during the desorption/ionization step by adding a reactant in the spray solvent.^19)^ This can enable the detection of molecules that are difficult to ionize. DESI is limited to relatively low spatial resolutions (50–150 μm). With the application of nano-DESI, higher spatial resolution is permitted. This modification allows a lateral resolution as low as ∼10 μm.^20)^

### Post-ionization

The ionization efficiency of MALDI is estimated to be <10^−4^ relative to generated neutrals,^21,22)^ or even significantly lower depending on the compound class, which limits the sensitivity of this technique as many molecules are not detected. A method to boost the MALDI ion yield is a post-ionization strategy. Various post-ionization strategies have been applied such as plasma ionization, laser-induced post-ionization, or photoionization.

Steven *et al.* used an atmospheric pressure MALDI MS ion source in transmission mode and incorporated a plasma device into the ion source for post-ionization. By using plasma in addition to the laser irradiation, there was a significant increase in the intensity of the drugs amiodarone, paclitaxel, and probucol. Furthermore, a murine brain was imaged as a proof-of-concept, which did not only show a significant enhancement of ion intensities, but also a shift in the dominant species ionized and detected with plasma post-ionization.

Alternatively, Soltwisch *et al.* used laser-induced post-ionization, also known as MALDI-2, for signal enhancement of lipids.^21)^ In MALDI-2, a second ultraviolet (UV) laser beam intercepts the plume of the matrix and analytes generated by the standard MALDI laser.^21,23)^ They showed an increase in signal intensities of lipids in mouse cerebellum, which are otherwise difficult to image by conventional MALDI MSI. Barré *et al.* also used MALDI-2 to enhance sensitivity and signal intensity of various pharmaceutical compounds.^22)^ In addition, it was shown that ibuprofen, which is not detectable with regular MALDI, was detectable as a radical cation using MALDI-2. It is hypothesized that the ionization mechanism in MALDI-2 involves resonant two-photon ionization of the matrix.^21)^ Nevertheless, a full understanding of the MALDI-2 ionization, just like the regular MALDI ionization process, remains elusive.^21,22,24)^

Aside from MALDI, also other ionization techniques can benefit from post-ionization. Liu *et al.* combined DESI with post-photoionization by using a portable krypton lamp for secondary ionization of the desorbed neutrals.^25)^ They noticed an increase in the intensity of nonpolar lipids and neutrals, including cholesterol and γ-aminobutyric acid (GABA), showing post-ionization could be a great alternative for chemical derivatization.

### LESA

LESA is not an ionization technology, but an ambient surface sampling technique that involves liquid extraction from the surface of a tissue section.^26)^ A small droplet of solvent is used to extract soluble analytes with a liquid microjunction. This is coupled with a nano-ESI source for ionization. Traditional LESA experiments have a pixel size limited by the diameter of the solvent droplet, which is usually 1 mm.^26,27)^ However, high-resolution LESA has been published reaching a spatial resolution of 400 μm.^28)^ Until recently, LESA was used for sensitive tissue profiling. Nevertheless, recent developments made it possible to also generate (low-resolution) images by sampling at multiple points across a tissue.^26)^ An enormous advantage of LESA is that it allows manipulation of the extracted material prior to ionization. Therefore, liquid-based separation such as micro-liquid chromatography (μLC) can be incorporated, allowing, for example, isomeric separation.^28)^

## MS SYSTEMS

Concerning MSI, the two most commonly used categories of mass spectrometers are time-of-flight (TOF) and Fourier transform MS (FTMS). The latter one includes both Orbitrap-MS and Fourier Transform Ion Cyclotron Resonance (FTICR) MS. TOF analyzers are widely used for imaging experiments, due to the good sensitivity, relatively low cost, and high-throughput.^29)^ High-throughput capabilities are opening new frontiers for MSI, as with these speeds, it is possible to image larger areas or a greater number of samples, which is critical for clinical studies or 3D-MSI.^10)^

In the ion source of the TOF-MS, the ion packet is generated and, after some delay, accelerated into the ion extraction region. The pulsed character of ion generation with MALDI perfectly combines with TOF analyzers.^13)^ There are two geometries of TOF analyzers commonly used, namely axial and orthogonal. The axial geometry includes both linear and reflectron TOF-MS configurations, however, orthogonal TOF (*o*-TOF) can also use reflectrons. Linear TOF-MS is often used for high mass range measurements. Reflectron TOF-MS uses an electrostatic ion mirror to compensate for the differences in kinetic energy obtained during desorption/ionization processes. This only works in combination with delayed extraction. Because of this kinetic energy compensation, the ion packet is more compact when it reaches the detector, resulting in a significant improvement of the mass resolution. However, using a reflectron is not convenient for high-mass analysis due to metastable decay.^1)^ The *o*-TOF geometry led to the introduction of a hybrid quadrupole mass analyzer with a TOF-MS (QTOF), which has a higher mass accuracy compared to the axial-TOF geometry because of the decoupling of mass analysis from the initial energy distributions created in the source. Additionally, orthogonal acceleration can converge spatial and energy dispersion in the accelerated direction. An additional advantage of the *o*-TOF geometry is the possibility of the utilization of continuous beams. In an imaging context that enables the use of DESI or other continuous beam based ionization technologies. However, the duty cycle is lower, which comes at the cost of sensitivity. When high sensitivity is needed in a targeted approach, multiple reaction monitoring (MRM) can be utilized by using a triple-quadrupole (QqQ) or ion trap mass spectrometer. Here, multiple compounds can be targeted simultaneously, and since MRM is specific for the precursor and product ions, it can be used to separate isobaric compounds with improved sensitivity.^30)^ Aside from ESI-based ionization methods, also MALDI-MRM-MS has shown to be a feasible alternative method for sensitive and selective analysis.^31,32)^ It is also possible to use TOF-MS systems for tandem-MS, namely with an axial TOF-TOF configuration. Here, the first TOF is used to select a precursor ion and the second TOF is used for fragment analysis. Nevertheless, the wide isolation window is a disadvantage of this configuration.

### High mass resolution

Although TOF analyzers are sensitive and fast, they have limited mass accuracy and resolving power. FTICR and Orbitrap are high-performance mass analyzers that are able to resolve isobaric species, which is typically beyond the capabilities of TOF analyzers. With an FTICR, the *m/z* of an ion is determined by its ion cyclotron resonance frequency in a uniform static magnetic field. Similarly, in an Orbitrap, the *m*/*z* of an ion is determined from its oscillation frequency in a radial logarithmic potential between the electrodes. As frequencies can be measured with higher precision, these instruments have a higher mass accuracy compared to TOF analyzers.^33,34)^

**Figure 1:**
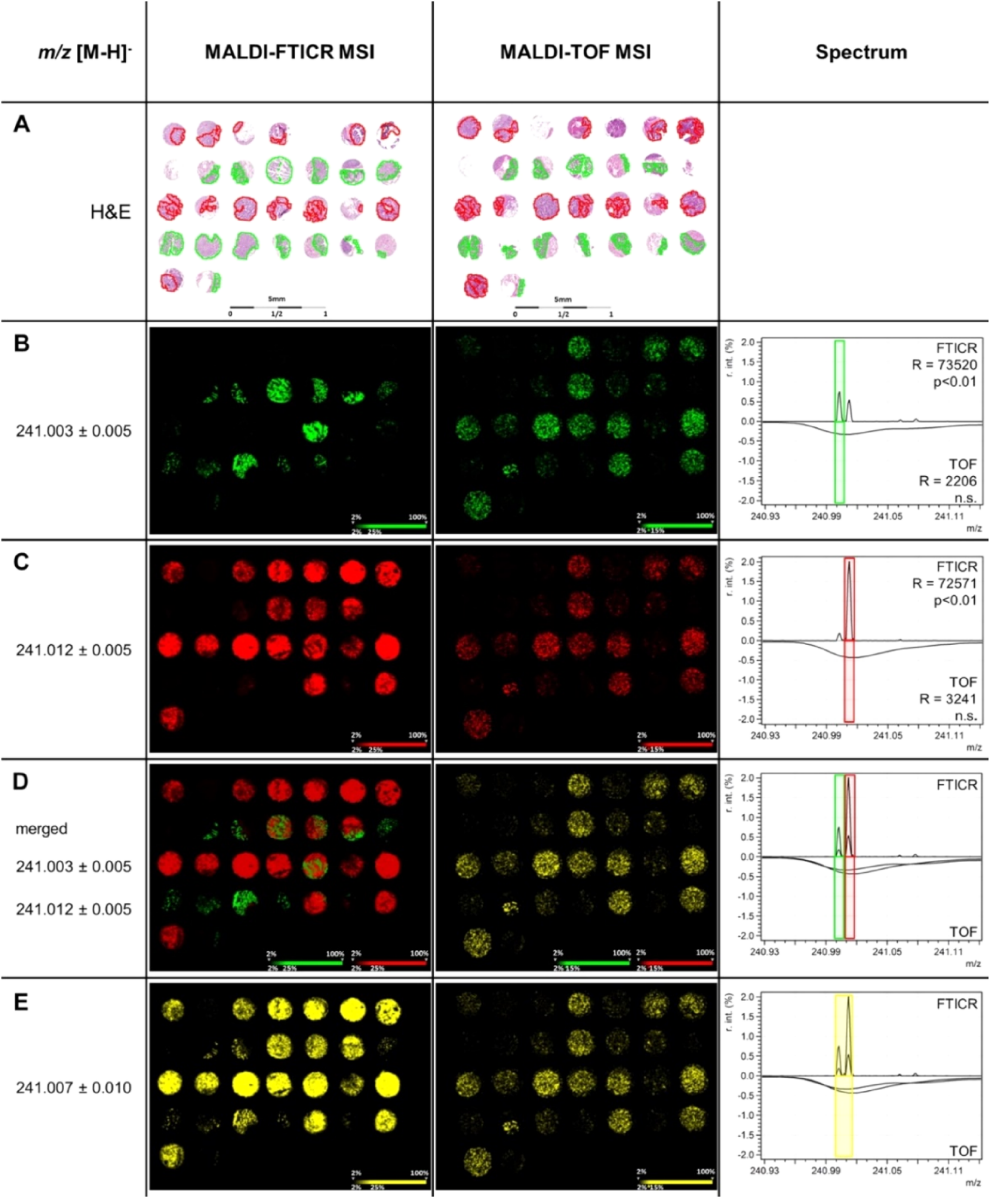
Fig. 1. (A) Hematoxylin and Eosin (H&E) stain of a colon tissue microarray with annotated normal epithelium regions (green) and tumor regions (red). Mass spectra and ion distribution maps show differences in significance levels and localization of the ions (B) *m*/*z* 241.003±0.005 (green) and (C) *m*/*z* 241.012±0.005 (red) with MALDI-FTICR and MALDI-TOF MSI. (D) In high-mass resolution imaging, the two analytes are clearly defined as different molecular components discriminating normal colon epithelium and tumor in MALDI-FTICR MSI. Imaging with lower resolution combined signals making it appear as a single peak. In the TOF image, a superimposition of green and red results in yellow demonstrating ion co-localization. (E) Simulation of the TOF image by the selection of a wider bin width combining both peaks (*m*/*z* 241.007±0.010) in FTICR spectrum. *Reprinted with permission from A. Buck, B. Balluf, A. Voss, R. Langer, H. Zitzelberger, M. Aichler, A. Walch, How Suitable is Matrix-Assisted Laser Desorption/Ionization-Time-of-Flight for Metabolite Imaging from Clinical Formalin-Fixed and Paraffin-Embedded Tissue Samples in Comparison to Matrix-Assisted Laser Desorption/Ionization-Fourier Transform Ion Cyclotron Resonance Mass Spectrometry?, Anal. Chem. (2016) 88, 10, 5281–5289. Copyright © 2016 American Chemical Society*.

Both mass accuracy and mass resolution are necessary to be able to make confident peak assignments. Mass accuracy, the ratio of the *m*/*z* measurement error to the true *m*/*z* in parts per million (ppm), is a measure of the precision of mass measurements. A mass accuracy of less than 3 ppm can, together with fragmentation spectra, assist in providing elemental composition or even provide information on its structure.^34)^ Without any fragmentation data, the accurate mass can function as a powerful filter for the identification of a compound. The mass resolution, the measure of the ability to separate two peaks with a slightly different *m*/*z*, defined as the full width at half maximum, defines the degree of chemical specificity. Especially with complex biological samples in mixture with matrix components, other compounds can overlap with the compounds of interest based on their mass. An example of this is shown in Fig. 1, where MALDI-FTICR MSI was able to separate peaks that are not resolved with MALDI-TOF MSI.

An efficient method to get structural information and the high-mass resolution data without increasing acquisition time is a multiplex MSI data acquisition method.^35,36)^ Here, data-dependent acquisition is used on a hybrid ion trap-Orbitrap instrument, where either the full MS is measured in the Orbitrap and the MS/MS in the adjacent pixel in the ion trap, or each raster step is split into spiral steps. This is done in parallel and independent from the Orbitrap. Precursors are automatically selected based on the previous FTMS scan. In the end, the full MS pixels can be extrapolated to visualize the molecular distributions. This makes it possible to gain structural information together with high-resolution MSI allowing making structural assignments more confident in the same amount of time while preserving the spatial data.

Although the strengths of high mass resolution systems, there are also some downsides. The first drawback of such a system is the data acquisition speed. MSI acquisitions on trapping-type instruments are fundamentally restricted by the scanning speed of the mass analyzer.^10)^ Therefore, the extended measurement times are making these analyzers less practical for analyzing large cohorts. The second drawback is the data size. High-mass resolution systems are producing files containing millions of data points. This presents a challenge in data handling and analyzing.^29)^

### Ion mobility spectrometry

A post-ionization separation technology that is often used in combination with MS is ion mobility spectrometry (IMS). IMS has proven to be an efficient method for the gas-phase separation of structural and stereoisomers, by separating ions based on their mass, charge, and collision cross-section. There are various variations of IMS, such as traveling wave ion mobility spectrometry (TWIMS) and Drift tube ion mobility spectrometry (DTIMS), that have been used for isomer separation and structure elucidation.^37, 38)^

## APPLICATIONS OF ISOMER IMAGING

As high-mass resolution only is not enough to separate isomeric compounds, various methods have been developed to improve isomer identification in imaging experiments. Here, different approaches on how to deal with these complexities in an imaging setting are discussed, with a focus on lipids, steroids, amino acids, and proteins.

### Lipids

Lipids are a diverse group of biomolecules that are involved in a wide range of biological processes, including being a key component of cellular membranes, cell signaling,^39)^ energy storage^40)^ and involvement in the regulation of metabolic pathways.^41)^ The functionality of a lipid is highly dependent on its molecular structure. Therefore, it is of importance to identify the complete lipid structure in order to understand their biological function.

There are five levels of identification of lipids: 1) lipid class, 2) the total number of carbons and double bonds in the acyl chains, 3) the number of carbons and double bonds per acyl chain, 4) the location on the lipid backbone where acyl chains are attached (*i.e.*, *sn*-position), and 5) the location and the stereochemistry of double bonds.^42)^ Standard low-energy collision-induced dissociation (CID) mass spectrometry tools can provide information at the first three levels. However, CID often does not reveal the more subtle structural characteristics such as *sn*-position and double bond position,^43,44)^ although it has been shown that information on the *sn*-position can also be achieved with CID for some lipid classes under the right conditions.^45)^ Recently, new approaches have been developed to tackle these limitations and allow more complete lipid structure determination, including ultraviolet photodissociation (UVPD), Paternò-Büchi (PB) reactions, ozone-induced dissociation (OzID), charge-remote fragmentation (CRF), high-field asymmetric waveform ion mobility spectrometry (FAIMS) and *meta*-chloroperoxybenzoic acid (*m*CPBA) epoxidation.

#### Ultraviolet photodissociation

**Figure 2:**
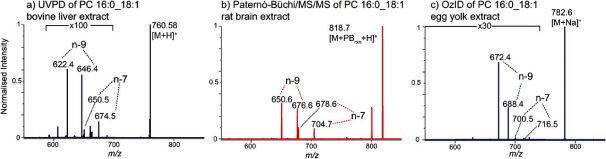
Fig. 2. Differentiation of PC 16 : 0_18 : 1 double bond isomers using various ion-activation strategies. (a) UVPD of a bovine liver extract, (b) PB-MS/MS from a rat brain extract and (c) OzID from an egg yolk extract. *Reprinted from T. Porta Siegel, K. Ekroos, S.R. Ellis, Reshaping Lipid Biochemistry by Pushing Barriers in Structural Lipidomics, Angew. Chem. Int. Ed. Engl. (2019) 58, 20, 6492–6501 with permission*.

Post-ionization with 193 nm UVPD is an ion activation method used for detailed structural characterization of lipid species.^46)^ This technique is capable of differentiating double bond positional isomers,^47)^ or, with a hybrid MS^3^ strategy, reveal the *sn*-position based on their relative abundance.^48)^ With UVPD, the carbon–carbon bonds adjacent to the double bond are cleaved, providing diagnostic pairs of fragments with a difference of 24 Dalton (Da)^46)^ (Fig. 2a). Absorption of a 193 nm photon results in higher energy excitation, which gives access to fragmentation pathways not observable with CID.^47)^ Due to the short activation period, UVPD enables fast acquisition making it suitable to connect on-line with MSI or LC-MS/MS.^47,49)^ The on-line coupling of UVPD with MSI was demonstrated by Klein *et al.*^47)^ Here, the coupling of DESI with UVPD was used for the characterization of phospholipid isomers in tissue sections. The presence of both phosphatidylcholine (PC) 16:0_18:1(Δ9) and PC 16:0_18:1(Δ11) isomers in human lymph node tissue containing thyroid cancer metastasis was confirmed by UVPD-MS. The ratio images unveiled spatial changes in the relative abundances of phospholipid double bond isomers between normal and diseased tissue.

#### Paternò–Büchi reaction

The PB reaction is a classical [2+2] photochemical cycloaddition reaction. Lipids are ionized from a solution containing acetone with ESI where UV irradiation of the plume at 254 nm is used to excite the carbonyl group within aldehydes and ketones.^50)^ This produces bi-radical intermediates, which subsequently react with the double bond yielding diagnostic fragments 26 Da apart specific for the double bond position^50,51)^ (Fig. 2b). Advantages of this reaction include the simple setup and no need for MS instrument modification.^50)^

Zhang *et al.* applied PB-MS/MS post-LC separation with hydrophilic interaction chromatography for the localization of double bond isomers in polar lipid extracts from human breast cancer tissue samples.^52)^ Here, acetone was directly employed in the mobile phase. The PB reaction was implemented immediately before ESI to preserve the retention time of lipid subclasses. A flow microreactor was developed to allow even exposure to UV irradiation for the reaction to take place. Twelve isomer pairs with significant changes in Δ9/Δ11 between normal and cancerous tissue samples were found for both PCs and phosphatidylethanolamines (PEs). Ma *et al.* also showed the application of PB-MS/MS after nano-ESI to pinpoint double bond locations in breast cancer tissues.^53)^ They found that the relative percent of ∆11 isomer for PC 16:1_18:1 showed no significant change between normal and cancerous breast tissues. However, they did find the ∆11 isomers of 18 : 1 to be significantly elevated in the breast cancer tissue.

Bednarik *et al.* found that acetone is not optimal for subsequent MALDI MSI analysis due to the large droplet size upon condensation of the vapor.^50)^ An on-tissue PB reaction using benzaldehyde vapor was performed to create a film of the reagent, after which the PB reaction was initiated using a UV lamp emitting at 254 nm. This off-line derivatization makes the technique compatible with commercial MALDI-MS/MS systems. Another off-line derivatization strategy has been published by Wäldchen *et al.*, who used benzophenone as PB reactive MALDI matrix.^54)^ Here, UV irradiation at 343 nm during the regular laser desorption/ionization process activates the reaction with unsaturated phospholipids. An alternative approach has been published by Tang *et al.*, who constructed a liquid microjunction surfaced sampling probe to extract lipids from tissue surface and couple the online PB reaction with ambient mass spectrometry.^55)^ This makes it possible to analyze directly from tissue and allows identification and relative quantitation of lipid double bond locations.

#### Ozone-induced dissociation

OzID is an ion activation technology using a gas-phase reaction between mass-selected ions and ozone vapor inside a mass spectrometer.^56–58)^ During this ozonolysis reaction, the ozone molecule reacts with the double bond and forms a primary ozonide. This primary ozonide is very unstable and therefore dissociates further into aldehyde and Criegee product ions. Each double bond produces an aldehyde and a Criegee ion, separated by 16 Da^39,58)^ (Fig. 2c). Another approach possible which features OzID is the sequential combination of CID and OzID. In these experiments, the CID product ions are isolated and subsequently, following the reaction with ozone, result in selective cleavage of the *sn*-2 acyl chain.^42,59,60)^ This makes OzID suitable for both assigning double bond positions and *sn*-positions.

Notable is the presence of sodium adducts in previously published papers. Driving the lipids to the sodiated adduct with *e.g.* sodium acetate has been found to be beneficial for the OzID, as the sodiated lipids have a higher yield of ozonolysis products compared to the protonated or potassiated lipids.^39,57,61)^ This is consistent among various publications, indicating that sodium has a faster reaction rate and suggesting the participation of the charge carrier, perhaps *via* chelation of the double bond, in the mechanism of the reaction.^56,58)^ It has also been found that the *trans* isomer reacts faster than the *cis* isomer.^58,62)^ Branching ratios in ozonolysis reactions are sensitive to the structure of the primary ozonide, which in turn is influenced by the double bond geometry.^39,58)^ However, since the product ions are the same, this distinction is based on the intensity. Also conjugated double bonds have shown to have a higher OzID efficiency than monounsaturated double bonds, which can be explained due to the influence of double bond conformation and metal-ion adduction.^41,58)^

**Figure 3:**
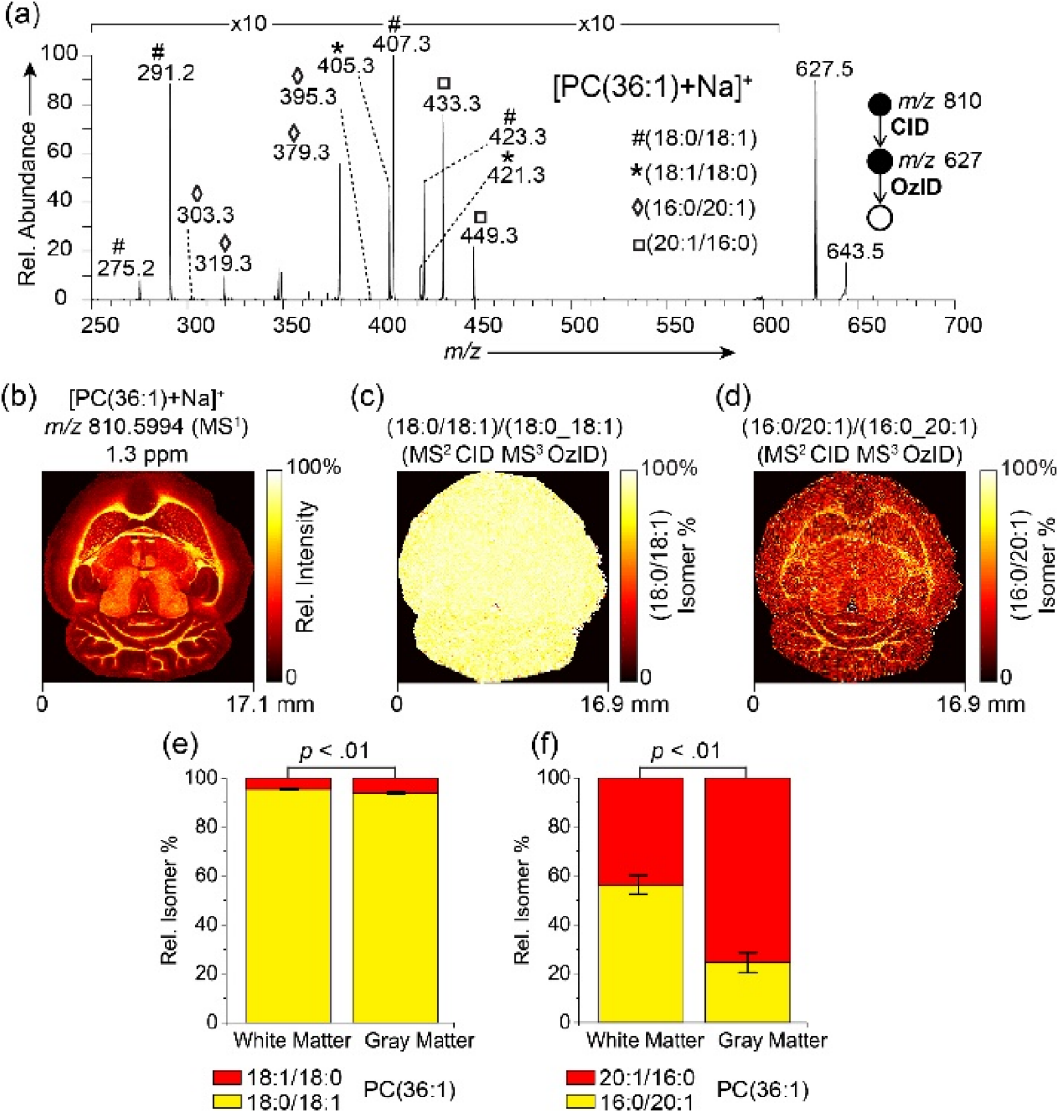
Fig. 3. (a) MALDI-CID/OzID spectrum of *m*/*z* 810, identified as [PC36 : 1)+Na]^+^ revealing the presence of four *sn*-positional isomers, namely PC(18 : 0/18 : 1), PC(18 : 1/18 : 0), PC(16 : 0/20 : 1) and PC(20 : 1/16 : 0). (b) Full-scan FTMS image of [PC(36 : 1)+Na]^+^. (c, d) The corresponding fractional distribution images of (c) PC(18 : 0/18 : 1) as a fraction of PC(18:0_18:1); and (d) PC(16 : 0/20 : 1) as a fraction of PC(16:0_20:1)-related ions. (e, f) Graphs with relative isomer percentages for (e) 18 : 0/18 : 1 and 18 : 1/18 : 0 isomers for PC(36 : 1) and (f) 16 : 0/20 : 1 and 20 : 1/16 : 0 isomers for PC(36 : 1) within the white and gray matter. *Adapted from M. R. L. Paine, B. L. J. Poad, G. B. Eijkel, D. L. Marshall, S. J. Blanksby, R. M. A Heeren, S. R. Ellis, Mass Spectrometry Imaging with Isomeric Resolution Enabled by Ozone-Induced Dissociation, Angew. Chem. Int. Ed. Engl. (2018) 57, 33, 10530–10534 with permission*.

Recently, M. Paine *et al.* published the combination of OzID with MSI, showing differential distributions of both double bond and *sn*-positional lipid isomers in brain tissue^61)^ (Fig. 3). Here, a MALDI source was coupled to a modified LTQ-Orbitrap Elite mass spectrometer to perform ozonolysis in the linear ion trap. An example was given of [PC(36 : 1)+Na]^+^, which showed the presence of four distinct *sn*-positional isomers (Fig. 3a). The summed distributions show an enrichment in the white matter (Fig. 3b). However, the fractional distribution images (Fig. 3c, d) and the isomer ratios graphs (Fig. 3e, f) show only a slight alteration in isomer ratios between white and gray matter of [PC(18 : 0/18 : 1)+Na]^+^ relative to [PC(18 : 1/18 : 0)+Na]^+^, but [PC(16 : 0/20 : 1)+Na]^+^ to be enriched in the white matter relative to isomeric [PC(20 : 1/16 : 0)+Na]^+^.

#### Charge-remote fragmentation

CRF is a type of gas-phase decomposition similar to gas-phase thermolysis, which occurs remotely from the charged site of a molecule.^63)^ The fixed charge is important to assure that CRF is the predominant reaction. With an unfixed charge, there can be competition between CRF and charge-driven reactions, resulting in spectra that are difficult to interpret. Under high-energy CID (HE-CID), CRF takes place and product ions are generated that allow elucidation of the double bond position and the *sn*-position of lipids.^64)^ The energy supplied by HE-CID is important for the reaction. Fast atom bombardment has mostly been used as ionization method for CRFs.^63)^ This concept of ionization is also exploited with SIMS. Additionally, since a long alkyl chain with a fixed charge always undergoes CRF, also ESI and MALDI can be used for ionization.

G. Fisher *et al.* showed that HE-CID in TOF-SIMS can be used to identify the double bond position.^65)^ They noted the effect of unsaturation in the spectra generated from fatty acid 18 : 0 and 18 : 1, stearic acid and oleic acid respectively, and confirmed this using erucamie, a polymer additive with a double bond at Δ13. Shimma *et al.* used CRF under HE-CID to locate the double bond location from PC 16 : 0_18 : 1 in mouse brain tissue using a MALDI-SpiralTOF-ReflectronTOF MS.^64)^ Based on the CRF peak pattern, it was confirmed the double bond was positioned at Δ9. Furthermore, they showed it was possible to distinguish between *sn*-positions based on the relative peak intensities, where *sn*-2, eliminated as a ketene, was more abundant compared to *sn*-1.

#### FAIMS

FAIMS is a variation on ion mobility spectrometry and is, compared to conventional IMS, a more orthogonal separation technique to MS. Because of this orthogonality, FAIMS tends to resolve isomers much better compared to linear IMS.^66,67)^ With FAIMS, gas-phase ions are separated based on differences in their mobilities in an electric field. By alternating high and low electric fields perpendicular to the ion travel path, a high-frequency asymmetric waveform, called the dispersion field, is employed. A compensation field counteracts the ion drift caused by the dispersion field to avoid ions colliding with the electrode walls.^68)^ This allows ions with specific mobilities to be transmitted into the mass spectrometer. Feider *et al.* successfully integrated DESI and a liquid microjunction surface sampling probe with a chip-based FAIMS device for imaging of biological tissue samples.^68)^ In addition, Bowman *et al.* demonstrated the power of FAIMS to separate lipid isomers and found a 75% success rate in resolving lipid isomer standards containing differences in *sn*-position, chain length, double bond position, and *cis*/*trans* isomerism, showing FAIMS is a powerful tool for rapid lipid isomer elucidation.^66)^

#### *m*CPBA epoxidation

Another approach exploited for lipid double bond isomer identification on tissues has been an epoxidation reaction with *m*CPBA. *m*CPBA is an oxidant that can be used to derivatize a double bond into an epoxide.^69)^ Subsequently, CID can be used for fragmentation, which will generate a diagnostic pair of fragments of 16 Da apart.^69,70)^ Feng *et al.* performed this reaction by mixing *m*CPBA in dichloromethane with yeast extract for direct infusion ESI-MS/MS analysis.^69)^ Kuo *et al.* performed *in situ* epoxidation by spraying tiny droplets of an *m*CPBA solution onto tissue sections before DESI-MSI analysis.^70)^ Intensity ratios of diagnostic ions were used to create a fractional distribution image of epoxy-fatty acid 18 : 1, showing an enriched level of the Δ11 isomer in the tumor region. The summed distribution of the lipid phosphatidylglycerol (PG) 18 : 1_16 : 0 was unable to distinguish this region.

### Steroid hormones

Steroid hormones play a major role in bodily functions such as maintenance of homeostasis during many biological events. Since steroid hormones are synthesized and released in the adrenal cortex, the gonads, and the placenta, the direct visualization of steroid hormones with MSI is one of the important analytical techniques without using immunohistochemistry to understand steroid-related diseases. However, visualization of steroid hormones is generally considered difficult due to the low polarity characteristic in MALDI or other ionization techniques.

#### Enhancing ionization

Cholesterol, a substrate of all steroid hormones, is difficult to ionize with ESI and MALDI due to its low proton affinity and low acidity.^71)^ Derivatization is a strategy that can be used to enhance its ionization. Patti *et al.* achieved imaging in mouse brain tissue using a nanostructure-initiator MS technique with the addition of NaCl or AgNO_3_ as a cation reagent.^72)^ Here, intact cholesterol was detected as silver adducts instead of molecular fragments from mouse brain tissue. Wu *et al.* also demonstrated an approach using reactive DESI by adding betaine aldehyde in the spray solvent to react with the alcohol group of the cholesterol.^71)^ This *in situ* derivatization enhanced the ionization of cholesterol and allowed to rapidly screen cholesterol in human serum and rat brain tissue. As an alternative to chemical derivatization, also post-ionization strategies can be used to enhance the ionization of nonpolar compounds such as cholesterol.^22,23,25)^

In addition to cholesterol, also imaging of other steroid hormones have been reported by enhancing the sensitivity. Recently, Cobice *et al.* have introduced Girard reagent T (GirT) as on-tissue derivatization and enabled the detection of steroids with MALDI.^73)^ GirT is known to be the most reactive with the ketone group at the C3 position of the A-ring of steroid hormones which is conjugated with a C4–5 double bond.^73)^ GirT hydrazine enhanced the ionization efficiency of steroids through cation charged trimethylamine and made it possible to detect cortisol in mouse brain tissue. Subsequently, testosterone in mouse testis and also triamcinolone acetonide in cartilage have been visualized.^5,73–75)^ Barré *et al.* showed the utility of GirT on-tissue derivatization also in the carbonyl group in the side chain at position 17 in comparison with 2,4-dinitrophenylhydrazine reagent.^74)^ Although many kinds of derivatization reagents have been developed in liquid chromatography based ESI-MS, the current study showed the best performance with GirT in MALDI MSI.

#### Tandem MS to separate structural isomers

**Figure 4:**
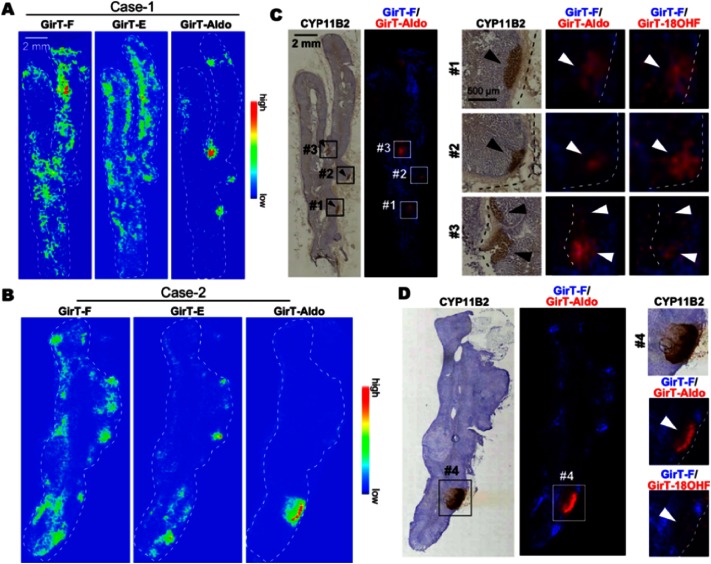
Fig. 4. (A, B) Distribution of GirT-steroids in healthy human adrenal glands. Whole (A, B, C, E) and enlarged (D, F) views of CYP11B2 immunoreactivity and detection of GirT-Cortisol (GirT-F, *m*/*z* 387.2), GirT-Cortisone (GirT-E, *m*/*z* 385.2), GirT-Aldosterone (GirT-Aldo, *m*/*z* 397.2), and GirT-18-hydroxycortisol (GirT-18-OHF, *m*/*z* 413.2) by MALDI MSI. *Reprinted with permission from E. Takeo, Y. Sugiura, T. Uemura, K. Nishimoto, M. Yasuda, et al., Tandem Mass Spectrometry Imaging Reveals Distinct Accumulation Patterns of Steroid Structural Isomers in Human Adrenal Glands, Anal. Chem. (2019) 91, 14, 8918–8925. Copyright © 2019 American Chemical Society*.

Steroid synthesis is simple in rodent adrenals. However, the human steroidogenesis pathway in the adrenal gland is more complex and some steroids provide the same chemical formula as its structural isomers. The existence of these structural isomers would interfere with the MSI results because the isomers detect as the same signal even using high-mass FTICR-MS imaging. Shimma *et al.* showed the potential of tandem MS using an ion-trap to discriminate testosterone from dehydroepiandrosterone in mouse testis.^5)^ Furthermore, Sugiura and Takeo *et al.* reported isomer selective imaging results of steroids by the combination of GirT derivatization and ion-trap tandem MS.^76,77)^ The authors currently propose MS^3^ imaging for GirT-derivatized steroid hormones and clearly visualized aldosterone, cortisol, cortisone, and other hybrid steroids in the adrenal gland (Fig. 4).

#### Ion mobility spectrometry

**Figure 5:**
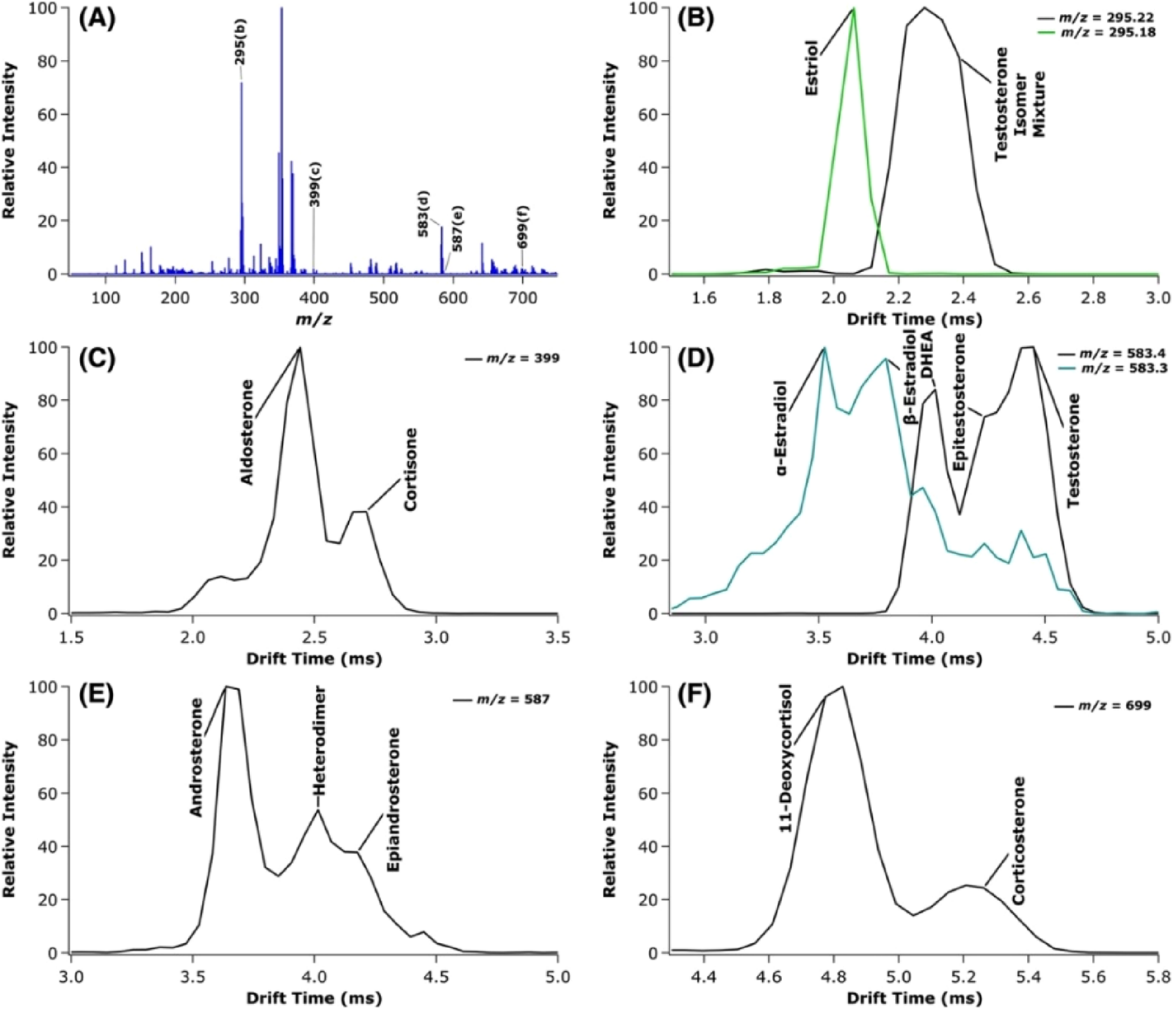
Fig. 5. Mass spectrum of a complex steroid mixture (A) with TWIMS-MS arrival time distributions at the indicated *m*/*z* values for the estriol and testosterone isomers as lithiated monomers at *m*/*z* 295 (B); aldosterone and cortisone as potassiated monomers at *m*/*z* 399 (C); potassiated estradiol dimers and lithiated testosterone isomer dimers at *m*/*z* 583 (D); androsterone and epiandrosterone as lithiated dimers at *m*/*z* 587 (E); and 11-deoxycortisol and corticosterone as lithiated dimers at *m*/*z* 699 (F). *Reprinted from A. L. Rister, T. L. Martin, and E. D. Dodds, Formation of multimeric steroid metal adducts and implications for isomer mixture separation by traveling wave ion mobility spectrometry, J. Mass Spectrom. (2019) 54, 5, 429–436 with permission.*.

IMS could be considered as another potential technique to separate steroidal isomers combined with MSI. Even though there are still no reports about steroid imaging using IMS, the utility in the separation of steroids has been reported.^78–82)^ In 2013, Ahonen *et al.* achieved separation of α/β-steroid isomers using TWIMS with *p*-toluenesulfonyl isocyanate derivatization. Derivatization was found to be effective for an increase in the collision cross-section and/or the strength of ion/molecule interactions with the drift gas.^78)^ Rister *et al.* also utilized TWIMS for underivatized structural isomers of steroid through the formation of metal adducted dimers or multimers with alkali metals.^81,82)^ Chouinard *et al.* used classical DTIMS to separate underivatized stereoisomers and structural isomers of steroids as metal adducted steroid dimers.^79,80)^ Only Rister *et al.* discussed the separation in mixture condition and mimetic sample as the real biological condition^81)^ (Fig. 5). Separation of stereoisomers, not only structural isomers, is an advantage of using IMS. We expect that these methods will be applied to biological tissue IMS-MSI in the near future.

### Amino acids

Amino acids are an important class of organic substances containing both amino and acid groups.^83)^ Although there are more than 300 amino acids in nature, only 20 of them are known as proteinogenic amino acids (α-amino acids). Both non-protein α-amino acids and non-α-amino acids play important roles in metabolism, therefore understanding of the distribution of amino acids can aid in the elucidation of their mechanism and the location of biological and metabolic processes.

#### Enhancing ionization

Visualization of amino acids in tissues by MSI has been difficult technically due to their low ionization efficiency and interference of the matrix peaks in low *m*/*z* range. To overcome the problems to visualize amino acids, many approaches using chemical derivatization were reported.^84–86)^ All reagents targeted the amino group of amino acids to introduce cationic charged ions to improve the ionization efficiency. Toue *et al.* reported the chemical derivatization into MALDI MSI of amino acids.^84)^ Here, they used *p*-*N*,*N*,*N*-trimethylammonioanilyl *N*′-hydroxysuccinimidyl carbamate iodide (TAHS) reagent was used at 5 mg/mL in acetonitrile and visualized 7 amino acid derivatives in colon cancer xenografts. Manier *et al.* precoated the tissue slide with 4-hydroxy-3-methoxycinnamaldehyde (CA) as a derivatization reagent,^86)^ and Shariatgorji *et al.* used pyrylium salts such as 2,4-diphenyl-pyranylium tetrafluoroborate (DPP-TFB).^85)^

**Figure 6:**
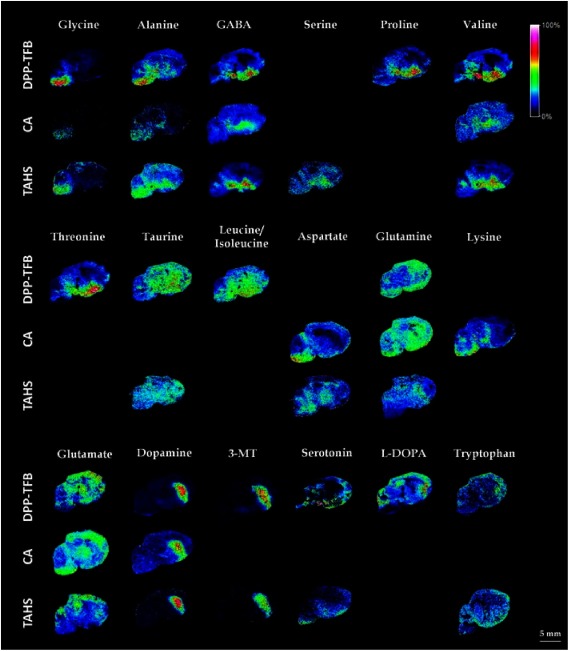
Fig. 6. MSI visualizations of derivatives of amino metabolites with TAHS, CA, and DPP-TFB in mouse brain sagittal sections with MALDI-FTICR in positive ion mode. *Reprinted from C. Esteve, E. A. Tolner, R. Shyti, A. M. van den Maagdenberg, and L. A. McDonnell*, *Mass spectrometry imaging of amino neurotransmitters: a comparison of derivatization methods and application in mouse brain tissue, Metabolomics (2016) 12, 30 with permission under CC BY 4.0. (*https://creativecommons.org/licenses/by/4.0/)

Esteve *et al.* reported the comparison among these three different derivatization reagents to improve the detection of amino metabolites and neurotransmitters.^87)^ They revealed that DPP-TFB dissolved in 100% methanol was found to be the best condition to obtain good spatial distribution with high ion intensity originated from charged *N*-alkyl pyridinium ions. However, in some specific cases like in the analysis of negatively charged ions, CA or TAHS reagent showed better results (Fig. 6). Esteve *et al.* concluded that the three derivatization reagents make up each other and the use of a combination of different reagents can be a valuable strategy for the detection of a wider range of amino metabolites. Alternatively, Liu *et al.* showed an increase in ion intensities of glutamine and glutamic acid, which could only be imaged by DESI with post-photoionization, showing post-ionization has the potential for targeting amino acids without chemical derivatization.^25)^

#### Tandem MS to separate structural isomers

These derivatization strategies can be applied to enhance the ionization of neurotransmitters such as GABA as well, which can also be classified as a γ-amino acid.^88)^ Manier *et al.* discussed the presence of structurally similar isobaric species for GABA and other neurotransmitters.^86)^ They showed the potential of combining CA derivatization and MS/MS (or MS^3^) measurement in the specification of analytes and visualized some neurotransmitters in the adrenal gland. However, they did not show the MSI image for GABA. On the other hand, Enomoto *et al.* did provide the visualization result of GABA with discrimination from structural isomers in *Drosophila melanogaster*.^89)^ Among the derivatization approach for amino acid analysis in MSI, only the CA reagent has the possibility to separate structural isomers. In MS/MS measurements, TAHS and DPP-TFB derivatives fragmented at the derivatized site and yielded derivatization-reagent derived peaks at *m*/*z* 177 for TAHS and *m*/*z* 232 for DPP-TFB for all amino acids.^85)^ Only CA derivatives provided analyte derived peaks.^86)^

#### Ion mobility spectrometry for enantiomers (L or D)

Most α-amino acids (except glycine) have chirality with one or two chiral center(s) where the central carbon has four different groups attached. These enantiomers often show completely different biological activities and therefore the chiral recognition of amino acids is important.^90–92)^ However, enantioselective MSI of amino acids has not been discussed nor achieved yet.

To accomplish the enantioselective MSI of amino acids, IMS, as previously described, would be one of the promising approaches since ion mobility is a post-ionization separation technique in the gas phase. Many studies have been performed to discriminate amino acid enantiomers using ion mobility mass spectrometry (Table 1),^93)^ and herein we will introduce four approaches that are using (i) chiral gas modifier in the buffer gas,^94)^ (ii) chiral selector with metal ions,^95–97)^ (iii) chiral selector without metal ions^98,99)^ or (iv) chemical derivatization.^100)^

**Table 1:** Table 1. Application of IMS in separation of amino acid enantiomers.

Authors	Year	The No. of separated amino acids	Separated amino acids	Type of IMS
Dwivedi *et al.*^94)^	2006	5	Met, Phe, Ser, Trp, Thr,	DTIMS
Mie *et al.*^96)^	2007	6	Arg, Lys, Val, Phe, Trp, Pro	FAIMS
Domalain *et al.*^95)^	2014	7	Arg, His, Glu, Tyr, Trp, Thr, Pro	TWIMS
Yu and Yao^97)^	2017	8	Arg, His, Met, Gln, Tyr, Phe, Trp, Thr	TWIMS
Zhang *et al.*^98, 99)^	2018	4	Phe, Trp, Cys, Pro	FAIMS
Perez-Miguez *et al.*^100)^	2019	17	Arg, Lys, His, Met, Val, Gln, Tyr, Phe, Ser, Trp, Asn, Ile, Leu, 2-Aminoadipic acid, pipecolic acid, selenomethionine, Orn	TIMS

**Figure 7:**
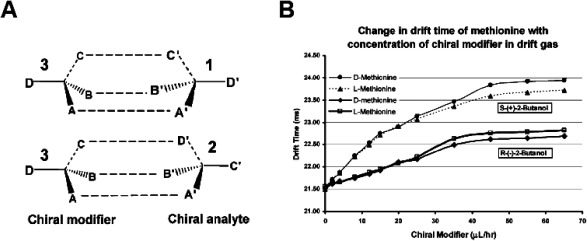
Fig. 7. (A) Schematic illustrating the three-point “Pirkle Rule” required for chiral recognition. Chiral IMS separation utilizes stereochemically different noncovalent interactions between the enantiomers (1 and 2) and the chiral modifier (3), (B) Effect of chirality and flow rate of the modifier on the arrival times of the methionine enantiomers. The figure shows retardation in mobility of methionine enantiomers with increasing concentration of either (*S*)-(+)-2-butanol or (*R*)-(−)-2-butanol as the chiral modifiers in nitrogen drift gas. Better separation of enantiomers was observed with (*S*)-(+)-2-butanol as the chiral modifier (separation factor of 1.01) as compared to (*R*)-(−)-2-butanol (separation factor of 1.006). *Reprinted with permission from P. Dwivedi, C. Wu, L. M. Matz, B. H. Clowers, W. F. Siems, H. H. Hill. Jr, Gas-phase chiral separations by ion mobility spectrometry, Anal. Chem. (2006) 78, 24, 8200–8206. Copyright © 2006 American Chemical Society*.

Dwivedi *et al.*^94)^ achieved IMS based separation of amino acid enantiomers using DTIMS in 2006. (*S*)-(+)-2-Butanol was injected into the nitrogen carrier gas as a chiral modifier, which provided an asymmetric chiral environment by forming diastereomeric cluster ions based on the Pirkle rule (Fig. 7A). Although the drift time of enantiomers is identical in pure nitrogen, five pairs of amino acid enantiomers were separated by doping chiral modifiers (Fig. 7B).

In 2007, Mie *et al.* reported separation of six amino acid enantiomers by forming diastereomeric complex ions [M^II^ (L-Ref)^2^(D/L-A)-H]^+^, where M^II^ is a metal ion, L-Ref is a chiral selector in its L-form and A is the analyte, and analyzing them with a home-built FAIMS system.^96)^ In the same manner, Domalain *et al.* achieved chiral amino acid analysis with an unmodified commercial TWIMS instrument.^95)^ Yu and Yao found that the formation of diastereomeric binuclear copper-bound tetrameric ions can improve chiral discrimination with TWIMS.^97)^ To avoid the formation and analysis of metal-bound trimers and higher-order multimers, in 2017, Zhang *et al.* directly analyzed diastereomeric proton bound complexes, that use modified amino acids with a butoxycarbonyl group (*e.g.* *N*-*tert*-butoxycarbonyl-*O*-benzyl-L-serine) as the chiral selector, with trapped ion mobility spectrometry (TIMS).^98,99)^

**Figure 8:**
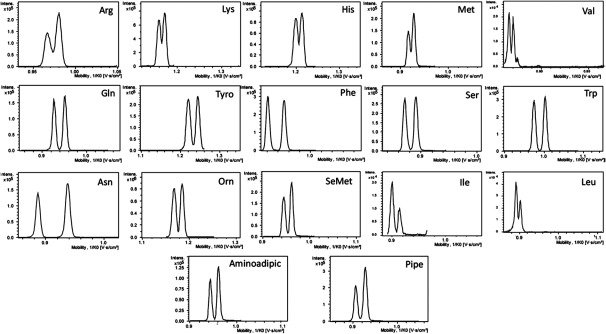
Fig. 8. TIMS-TOF-MS of 17 FLEC-amino acids using conditions optimized for each amino acid. *Reprinted from R. Perez-Miguez, B. Bruyneel, M. Castro-Puyana, M. L. Marina, G. W. Somsen, E. Dominguez-Vega, Chiral Discrimination of DL-Amino Acids by Trapped Ion Mobility Spectrometry after Derivatization with (+)-1-(9-Fluorenyl)ethyl Chloroformate, Anal. Chem. (2019) 91, 5, 3277–3285 with permission. Copyright © 2019 American Chemical Society*.

However, these approaches require specific reference compounds for each amino acid and the application was limited. In 2019, Perez-Miguez *et al.* newly proposed an alternative and generic approach for chiral separation of amino acids by employing TIMS and forming diastereomers through chemical derivatization with a chiral reagent ((+)-1-(9-fluorenyl)ethyl chloroformate (FLEC)).^100)^ With the addition of an alkali metal ion of sodium, separation of the FLEC diastereomers of 17 amino acids was achieved (Fig. 8).

### Protein structural analysis, isomers, and imaging

The study of the spatial distribution of proteins at tissue surfaces is crucial in the understanding, diagnosis, and treatment of many diseases. Targeted protein imaging strategies using antibodies, such as immunohistochemistry, are widely used in the pathology community and provide a very specific, sensitive and high-resolution image of their distribution. Multiplexed targeted protein imaging studies have become available in recent years that allow researchers to study several tens of different protein distributions simultaneously using a labeled antibody approach.^101)^ Label-free imaging of intact proteins can be achieved with imaging MS when a non-targeted strategy is required. It is often used as a discovery tool to investigate tissue-specific local proteome differences. This approach, often performed with high throughput MALDI-TOF MSI systems in linear mode, is complementary to the antibody-based targeted methods. Most of the intact protein MSI studies use the protein patterns to distinguish different cell types on tissue,^102)^ to trace the progression of a disease or to determine an effect of a drug treatment. Several research groups have investigated the use of MALDI MSI for the direct analysis of proteins from plant materials.^103)^ While it has become evident that intact protein imaging and screening has added value in tissue typing and surface imaging several challenges remain. In particular protein identification, the examination of local PTMs and the related proteoform identification, protein conformational studies and protein complex investigation directly from a biological surface are key to true spatial proteomics. All these require an investigation of protein structure at different levels, from primary structure to the local quaternary structure of a protein complex. Therefore, the true challenge in protein imaging MS is a structural challenge. Researchers have been pushing the boundaries to obtain improved levels of protein structural information over the last decades. Here, we will concisely discuss some of the solutions that offer the potential to provide more detailed insight in protein structure.

#### Protein structure determination

The amino acid sequence or primary structure of a protein can be readily determined by fragmenting the protein, either *via* digestion (bottom-up) or MS/MS (top-down). It is common practice in proteomics and results in thousands of protein identities from tissue extracts or other homogenized biological samples. The combined use of LC separations and high-performance MS has been an enabling technology in this field. MALDI-TOF MSI has three significant disadvantages when compared to proteomics. Firstly, it lacks the separation technologies, which results in significant ion suppression. Secondly, the mass resolving power of a linear TOF-MS system does not provide enough information to identify a protein. Lastly, the *m/*z range is limited as MALDI creates predominantly singly charged ions. This poses a challenge for researchers with an interest beyond protein-based tissue classification and that require protein identities. More analytical information is needed and a number of strategies have been developed to provide detailed local primary structural information. The added analytical information on protein structure is currently generated in three different manners: 1) the addition molecular resolving power, 2) the addition of ion mobility based structural separation and 3) the addition of LC to the MSI workflow.

Local primary protein structural elucidation in an imaging MS experiment is performed in a bottom-up or a top-down approach. Bottom-up protein imaging requires the sample surface to be proteolytically digested, typically using trypsin. This results in a distribution of tryptic peptides on the surface that are identified through peptide mass fingerprinting or local tandem mass spectrometry with MALDI MSI. The added advantage of this approach the ability to image formalin-fixed paraffin-embedded tissue as the digestion breaks protein cross-links that were generated in the fixation process.^104)^ Additional analytical information in this approach, as generated by any of the three approaches indicated above, improves the number of peptide sequences that can be identified.^105–107)^ Tryptic peptide-based protein identification has demonstrated its usefulness but is still limited in structural identification capabilities. The identification of post-translational modifications remains challenging, mainly due to the lack of suitable targeted on-tissue enrichment protocols for specific PTMs such as phosphopeptides. Multimodal strategies using in different combinations of enzymatic digestion strategies offer promise to address this particular challenge.^108)^ The lack of chromatographic separation in most applications of bottom-up proteomics causes substantial ion suppression and results in only a limited number of high abundant proteins identified.

Multimodal bottom-up strategies that take advantage of imaging mass spectrometry are gaining popularity to increase the molecular information content obtained from a single tissue. This field is often referred to as tissue microproteomics. The combination of MALDI-based tissue imaging with laser capture microdissection, followed by protein extraction and bottom-up LC-MS enabled proteomics of the selected regional tissue extracts provides extended structural identification possibilities. It allows the use of all liquid enrichment and separation technologies commonly employed in proteomics.^109–111)^ The molecular images obtained with MALDI MSI are deployed to guide the selection of interesting region for further analysis. This enables the identification of 1000 or more proteins from a selected tissue region. The proteins identified can be further validated through the use of combined and anatomically co-registered MALDI MSI and immunohistochemical staining on adjacent tissue sections.

**Figure 9:**
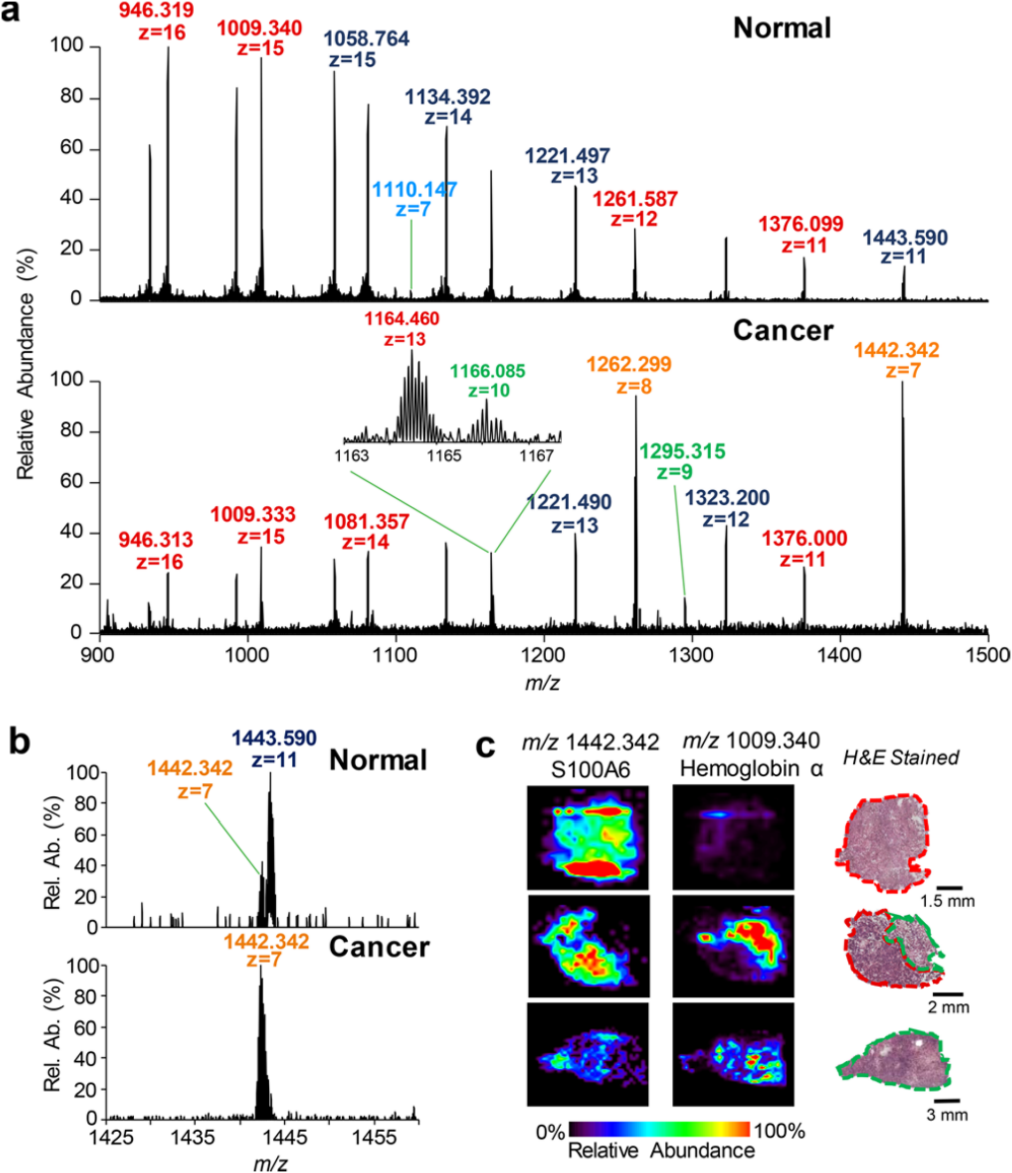
Fig. 9. Protein DESI-MS spectra of normal and high-grade serous ovarian cancer ovarian tumor with optimized FAIMS parameters. Mass spectra are averages of 10 scans. Different charge states of the same protein species are denoted by same-colored labels. (b) Zoomed in mass spectra of normal and high-grade serous ovarian cancer samples, highlighting the observance of *m/z* 1442.339, identified as S100A6, within the cancer tissue. (c) DESI-MS ion images obtained from normal, pure cancer, and mixed normal/cancer ovarian tissue sections. Ion images are on the same scale. H&E stained images are of a serial ovarian tissue sections as protein conditions are not histologically compatible. *Reprinted from K. Y. Garza, C. L. Feider, D. R. Klein, J. A. Rosenberg, J. S. Brodbelt, L. S. Eberlin, Desorption Electrospray Ionization Mass Spectrometry Imaging of Proteins Directly from Biological Tissue Sections, Anal. Chem. (2018), 90, 13, 7785–7789 with permission. Copyright © 2018 American Chemical Society*.

One area of strong development in imaging mass spectrometry is the application of top-down MSI. Top-down imaging refers to the absence of a proteolytic digestion protocol. Intact protein analysis is performed directly on tissue and a primary structural analysis is conducted using fragmentation technologies available inside the mass spectrometer. A substantial amount of work has been devoted to high-resolution intact protein imaging using a MALDI-FTICR-MS approach. This approached is challenged by the lack of fragmentation possibilities that provide the required sequence ions. This is directly related to the fact that MALDI predominantly produces singly charged ions and the large degrees of ro-vibrational freedom in these high molecular weight ions reduces the chance of a single bond breaking. An alternative approach is the use of different desorption and ionization methods that offer the possibility to locally generate multiply charged molecules from a tissue surface such as laser ablation electrospray ionization (LAESI) and DESI. LAESI has demonstrated to be able to generate multiply charged protein ions of high abundant proteins from tissue surface that can be identified with infrared multiphoton dissociation and electron capture dissociation.^112)^ The technique lacks the sensitivity to obtain high spatial resolution images of low abundant proteins and predominantly shows high abundant species. Developments in mass spectrometric sensitivity, improvements in the ionization cross-section and ion capture are the potential road towards a useful application of this technology in tissue imaging. An alternative approach to the study of multiply charged proteins from tissue surfaces is the use of DESI. Initially, researchers focused on the application of DESI to liquid surfaces which allowed the analysis of proteins and even non-covalently bound protein complexes.^113)^ Recently, the application of DESI combined with FAIMS for intact protein imaging from tissue was demonstrated to be able to distinguish healthy from cancerous tissue (Fig. 9).^114)^ UVPD and CID were employed for the top-down identification of the multiply charged protein ions desorbed from the tissue surface. Interestingly, abundant proteins, similar to those observed with intact protein MALDI-TOF MSI, were found when this approach was employed for the analysis of a coronal mouse brain section. Some of the recently published best practices and benchmarks for intact protein analysis for top-down mass spectrometry and the decision tree presented need to be translated to selected top-down MSI workflows.^115)^

#### Higher-order protein structure determination

The majority of MS-based tissue imaging focusses on the visualization and identification of primary protein structures. Few approaches exist that take on the challenge of visualizing higher order protein structures. Secondary protein structural elements such as alpha helices, beta sheets, beta turns and omega loops are difficult to elucidate with a mass spectrometric approach. Commonly circular dichroism, infrared spectroscopy or nuclear magnetic resonance are employed to determine the secondary structure, but all lack the molecular specificity mass spectrometry offers. Mass spectrometry offers structural investigation possibilities such as hydrogen-deuterium exchange, electron capture/transfer dissociation and chemical cross-linking that can provide limited secondary structural information. These technologies have not or only limitedly been deployed for imaging purposes. DESI and LAESI, as described in the previous section, are two technologies that, in combination with the structural identification technologies have the potential to distinguish protein isomers that differ only in their secondary structure in the future.

Tertiary structure or protein folding is the next level of structural detail that, in MS, results in ions with identical molecular weight but different folding structures. Several ion mobility methods exist that can distinguish isomeric molecules based on their collision cross-section. MALDI MSI has extensively been coupled to ion mobility separation using TWIMS^105)^ as well as many other ion mobility technologies (TIMS, FAIMS and a number of others) that have been discussed in an extensive review by McLean *et al.*^116)^ MALDI-IMS-MSI has demonstrated its usefulness for tryptic peptides and *N*-glycans up to a typical mass range of approximately 5,000 *m*/*z*. Intact proteins have rarely been investigated with this approach. This is a direct result of the fact that MALDI exclusively produces singly charged species and most common ion mobility instruments have a limited *m*/*z* range that precluded the analysis of species over 5,000 *m*/*z*. The use of ambient desorption and ionization techniques can, to some extent, overcome this because of collisional cooling or the capability to form multiply charged ions. Ambient ionization mass spectrometry offers a variety of sampling and ionization techniques. Only a small subset of these have proven suitable for intact protein analysis from thin tissue sections as discussed in the previous section. These ambient technologies have the added advantage that they allow ion mobility separation in the imaging workflow^117)^ and hence offer local tertiary protein structural information.

**Figure 10:**
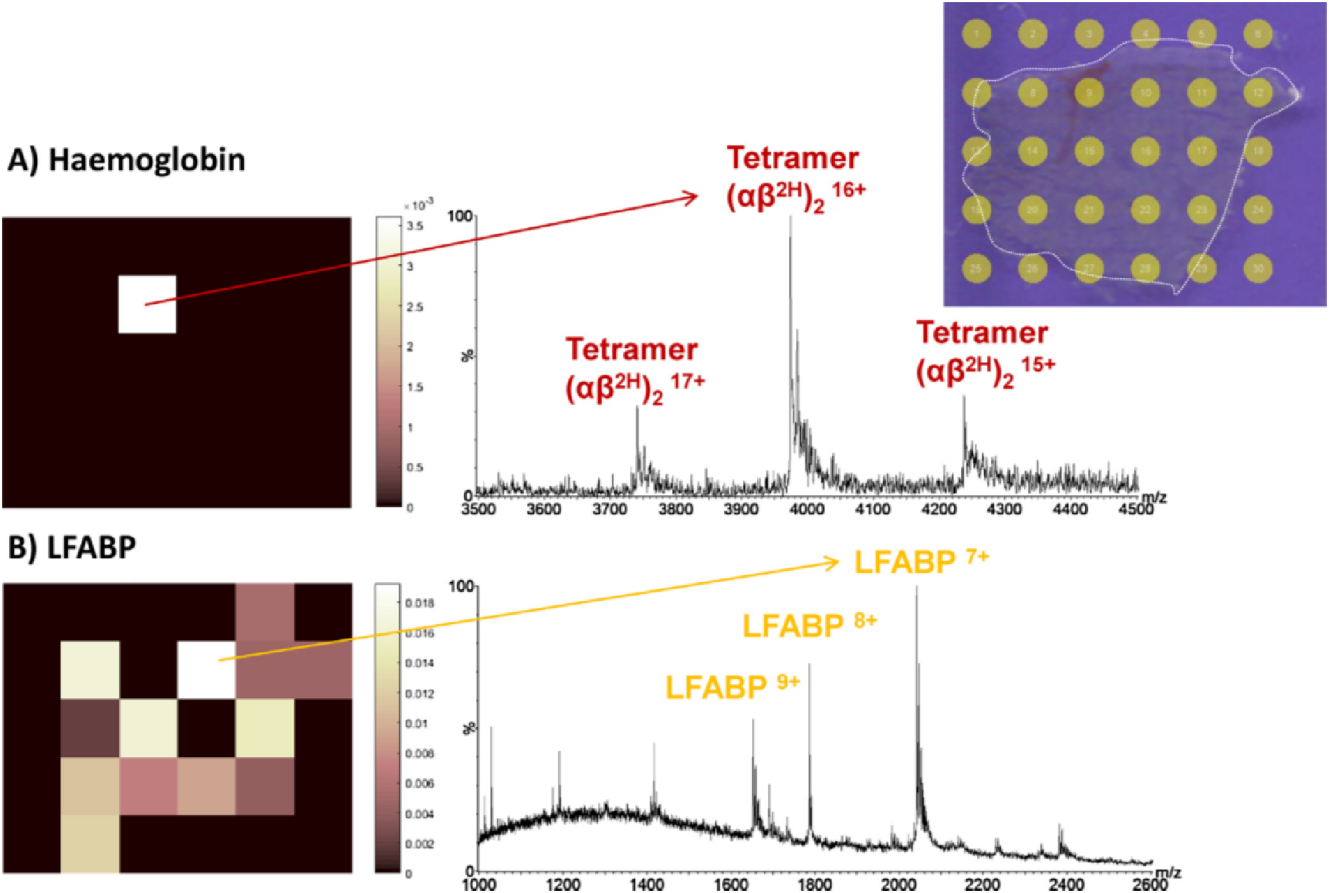
Fig. 10. Native LESA MS imaging of mouse liver. A) Ion image shown of *m/z* 3970 (16+, 64414 Da, (αβ^2H^)_2_ and the mass spectrum acquired at location (2,3) showing detection of the hemoglobin tetramer in the 15+, 16+ and 17+ charge states. B) Ion image of *m/z* 2039 (7+, 14264 Da, liver fatty acid binding protein (LFABP) and the mass spectrum acquired at location (2,4) showing detection of LFABP in 7+, 8+ and 9+ charge states. *Reprinted from R. L. Griffiths, E. K. Sisley, A. F. Lopez-Clavijo, A. L. Simmonds, I. B. Styles, H. J. Cooper, Native mass spectrometry imaging of intact proteins and protein complexes in thin tissue sections, International Journal of Mass Spectrometry (2019), 437, 23–29 with permission under CC BY 4.0.* (https://creativecommons.org/licenses/by/4.0/) (https://doi.org/10.1016/j.ijms.2017.10.009)

Quaternary structure, the number, and arrangement of multiple folded protein subunits in a multi-subunit complex is the ultimate level of structural isomeric analysis to be derived directly from tissue. The number of structural isomer possibilities at the quaternary (and tertiary) level is astounding and poses an enormous analytical challenge. The challenge is to keep the multi-subunit complex intact and unaltered during desorption and ionization. This is a common practice in the field of native mass spectrometry where purified protein complexes are dissolved in MS-compatible volatile buffer solution and routinely analyzed by electrospray MS. A complex tissue matrix is a completely different challenge and local desorption, needed to obtain an image, and requires local energy input that might disrupt the protein complex under study. One solution is the application of liquid microjunction extraction that has proven suitable for intact protein complex analysis from thin tissue sections.^118)^ In their work, Cooper *et al.* demonstrated the ability to use LESA to visualize the distribution of a native hemoglobin tetramer complex from a mouse liver (Fig. 10). While the image resolution is limited compared to other MSI technologies, the potential for native protein complex analysis from tissue surfaces is large.

#### Alternative approaches for protein structural analysis

The new gold standard in protein structural elucidation is the use of 3D cryo transmission electron microscopy (Cryo-TEM) in structural biology. It has become possible to identify even individual amino acids with resolutions in the low Angstrom range. The use of single-particle analysis enables the elucidation of the structure of large, native protein complexes. The contrast in Cryo-TEM offers morphological identification (and visualization) but lacks (bio-) chemical insights. The combination of TEM with MS-based approaches is evident and many researchers in structural biology pursue that approach. Sample purification, isolation, and cryo-preparation are still challenges for many proteins studies. Moreover, the single-particle studies lack the biological environment of the protein. Imaging mass spectrometry is highly complementary as it offers detailed insight in protein structure as discussed above. It is evident that one single technology alone will not provide all the pieces of information needed to understand the structure–function relationship. A significant effort will be placed on alternative hybrid or complementary tissue imaging strategies. A good example is the emergence of the use of combined MSI and top-down proteomics.^119)^ In these studies, MSI images are complemented with a tissue extraction and top-down analysis for protein identification. Other studies use images to guide or direct either laser capture microdissection^110)^ or liquid microjunction based sampling for follow-up analysis.^107)^ Structural imaging of protein isomers in tissue is still in its nascent state. It is clear that a substantial amount of work still is needed before it is possible to sensitively visualize the distribution of many proteoforms directly from a complex tissue surface. Image-guided tissue selection followed by high-end liquid-based proteomics currently offers the best opportunities, but with many novel and innovative technologies developed in intact protein mass spectrometry, a breakthrough might lurk around the corner.

## CONCLUSION AND PERSPECTIVES

In this review, we have discussed several technological innovations to improve the detailed identification of various compounds classes. Although many advances are still mainly a proof-of-concept, MSI with isomeric resolution demonstrated to have great potential for a better understanding of biological processes that otherwise would have remained unknown. These novel strategies are a great drive towards more complete structural characterization. Nevertheless, there are still limitations to overcome and further development is needed to enable total structural identification. Many methods are MS/MS based, meaning that only a select number of isomers can be analyzed in a given MSI experiment. In order to push structure elucidation to a greater extent, hybrid or complementary technologies could come to use such as a combination of the previously discussed approaches or by the use of complementary technologies. This could aid in unraveling the biological function or involvement in the biological processes of each isomer.

## Notes

Mass Spectrom (Tokyo) 2019; 8(1): A0078
