# Supplementary material for: Imaging Isomers on a Biological Surface: A Review
Source: Mass Spectrom (Tokyo). 2019 Dec 27;8(1):A0078. doi: 10.5702/massspectrometry.A0078 (PMC7035452; doi:10.5702/massspectrometry.A0078)
Supplement: Supplementary Data [file massspectrometry-8-1-A0078-s001.pdf]

Abbreviation	Definition
CA	4-hydroxy-3-methoxycinnamaldehyde
CID	Collision-induced dissociation
CRF	Charge-remote fragmentation
Da	Dalton
DESI	Desorption electrospray ionization
DPP-TFB	2,4-diphenyl-pyranylium tetrafluoroborate
DTIMS	Drift tube ion mobility spectrometry
ESI	Electrospray ionization
FAIMS	High-field asymmetric waveform ion mobility spectrometry
FLEC	(+)-1-(9-fluorenyl)ethyl chloroformate
FTICR	Fourier transform ion cyclotron resonance
FTMS	Fourier transform mass spectrometry
GABA	γ-Aminobutyric acid
GirT	Girard reagent T
H&E	Hematoxylin and Eosin
HE-CID	High-energy CID
IMS	Ion mobility spectrometry
LAESI	Laser ablation electrospray ionization
LC	Liquid chromatography
LESA	Liquid extraction surface analysis
MALDI	Matrix-assisted laser desorption/ionization
MALDI-2	Laser-induced post-ionization
*m*CPBA	*Meta*-chloroperoxybenzoic acid
MRM	Multiple reaction monitoring
MS	Mass spectrometry
MS/MS	Tandem mass spectrometry
MS^3	Triple tandem mass spectrometry
MSI	Mass spectrometry imaging
o-TOF	Orthogonal time-of-flight
OzID	Ozone-induced dissociation
PB	Paternò-büchi
PC	Phosphatidylcholine
PE	Phosphatidylethanolamine
PG	Phosphatidylglycerol
ppm	Parts per million
PTM	Post-translational modification
QqQ	Triple-quadrupole
QTOF	Hybrid quadrupole mass analyzer with a TOF
SIMS	Secondary ion mass spectrometry
TAHS	p-*N,N,N*-trimethylammonioanilyl *N'*-hydroxysuccinimidyl carbamate iodide
TEM	Transmission electron microscopy
TIMS	Trapped ion mobility spectrometry
TOF	Time-of-flight
TWIMS	Traveling wave ion mobility spectrometry
UV	Ultraviolet
UVPD	Ultraviolet photodissociation
